# Does previous sickness absence affect work participation after vocational labour market training? A difference-in-differences propensity score matching approach

**DOI:** 10.1093/eurpub/ckad154

**Published:** 2023-08-26

**Authors:** Laura Salonen, Svetlana Solovieva, Antti Kauhanen, Elli Hartikainen, Eira Viikari-Juntura, Taina Leinonen

**Affiliations:** Finnish Institute of Occupational Health, Helsinki, Finland; Finnish Institute of Occupational Health, Helsinki, Finland; Etla Economic Research, Helsinki, Finland; Finnish Institute of Occupational Health, Helsinki, Finland; Finnish Institute of Occupational Health, Helsinki, Finland; Finnish Institute of Occupational Health, Helsinki, Finland

## Abstract

**Background:**

Reduced work ability is relatively common among job seekers and it can hinder future labour market attachment. A commonly used measure to increase employability is the use of active labour market programmes. While vocational labour market training (LMT) has been shown to be an effective way to increase work participation among job seekers, there is still uncertainty about how LMT works in different population groups, for example, among persons with a work disability history.

**Methods:**

We used nationally representative Finnish register data on 16 062 LMT participants in 2008–2015 aged 25–59 with a history of sickness absence (SA) and propensity score matched participants without such history. For matching, we used information on sociodemographic and work-related factors. We used difference-in-differences analysis to investigate the differential changes in work participation before and after LMT between those with and those without SA history.

**Results:**

Having a history of SA was associated with a lower gain in work participation after LMT, but the magnitude varied by sex, employment history and follow-up time. In women, having a history of SA contributed to 3.9–6.2 percentage points smaller increase in work participation 1–3 years after LMT, depending on the employment history. In men, the corresponding numbers were 2.0–4.3 percentage points. The results were more pronounced if the SA was due to mental disorders.

**Conclusions:**

The results indicate that work disability, especially due to mental disorders, can hamper work participation after LMT and should be considered when planning employment-enhancing measures for job seekers.

## Introduction

Work disabilities and health problems are common among the unemployed,^1–3^ but they can remain unrecognized in health care and employment services,^2^^,^^4^^,^^5^ and unemployment may function as a hidden way of being absent from work due to work disability. These hidden work disabilities can hinder future employment possibilities and the effectiveness of active labour market programmes (ALMPs).

The most effective ALMPs in terms of later work participation are especially those that increase human capital, such as vocational labour market training (LMT).^6–11^ Meta-analyses and review articles^4–8^ have shown that the impact of LMT on employment outcomes is relatively strong and positive across European countries. Job seekers participating in LMT differ from other unemployed which can partially explain the success of these programmes. Participation in LMT is more common among younger men, secondary or tertiary educated, and among those with a more favourable employment history,^12–14^ compared to participants in other ALMPs. However, women, over 50 years old, and those who participate in longer training seem to benefit more from LMT in terms of later employment.^12–14^

To date, little is known about the role of work disability on LMT participation and the subsequent labour market outcomes. However, almost 30% of the LMT participants have been shown to have a work disability background.^15^ A Danish study found positive employment effects of ALMPs among sick-listed workers, but the study did not have a non-sick-listed comparison group.^16^ A Finnish study found that those with previous work disability were more often employed before LMT, but also experienced a stronger decline in their work participation after the training.^15^ Furthermore, work participation after LMT is likely affected by the diagnosis of work disability. While no studies exist on the association between diagnostic history and work participation among ALMP participants, studies have consistently shown that especially mental disorders are generally associated with poorer labour market attachment.^17–19^

To the best of our knowledge, this is the first study assessing the contribution of previous work disability on work participation after an LMT or any ALMP. In this study, utilizing nationally representative Finnish register data on individuals who participated in LMT in 2008–2015, we aimed to assess whether having a history of sickness absence (SA) affects work participation after LMT while accounting for the employment history. To reduce the selection bias in having a history of SA, we used propensity score (PS) matching. We used a difference-in-differences (DID) approach to assess the differential changes in work participation before and after LMT between those without and those with SA history due to all causes, mental disorders and other diagnoses. The analysis was stratified by sex and employment history.

## Methods

### Data sources and study population

We used a 70% random sample of the working-age population living in Finland on 31 December 2007. The data consisted of longitudinal individual-level information both prospectively and retrospectively. We obtained information on episodes of employment, unemployment, earnings-related retirement and vocational rehabilitation from the Finnish Centre for Pensions, on episodes of compensated sickness absence (SA) and national pensions from the Finnish Social Insurance Institution, and on sociodemographic factors, work-related factors and employment services including LMT from Statistics Finland. Register linkage was authorized by Statistics Finland (permission TK/141/07.03.00/2022-2) and Findata (permission Dnro THL/6341/14.06.00/2021).

In this study, we included job seekers (i.e. unemployed and individuals about to become unemployed) aged 25–59 years who started vocational labour market training (LMT) between January 2008 and December 2015 and finished it by the end of 2015. Incident LMT was determined as the first episode occurring in one of these years. The participant could have participated in other ALMPs as well.

We excluded individuals who received old-age pension, unemployment pension or disability pension three years before the training because they are unlikely to return to work. Only Finnish-born Finns were included in this study as immigrants are more likely to participate in other types of labour market programmes. To ensure a full follow-up time, we excluded individuals who were not living in Finland three years before and three years after the LMT. Moreover, to ensure that we have an employed population with occupational information at least from one relatively recent time point, we excluded those who did not have any information on their occupational status, industrial sector, or employer sector over a period of seven years before the LMT. The eligible study population before matching included 75 364 individuals.

### Vocational labour market training in Finland

In Finland, LMT is provided as a part of nationwide public employment services. LMT can be organized in many forms, ranging from short courses to the completion of a degree. Generally, job seekers who are unemployed or about to become unemployed and are at least 20 years old can be admitted to LMT. Participants’ health, motivation, prior training, work experience and suitability based on skills and characteristics required by the field in question, are assessed by a team consisting of experts of employment and economic development services and a representative of the training provider. Every year around 8% of job seekers apply for LMT (an average of 90 000 yearly applications) and around half of them are accepted. Of the accepted, most finish their training. There has been a decreasing trend since 2008 in the number of applications mainly due to economic fluctuations, legislative changes and changes related to LMT and other ALMPs.^13^

### Previous sickness absence

In Finland, all permanent residents aged 16–67 are entitled to sickness allowance. During the first 10 days (weekdays including Saturdays) there is a waiting period that is covered by the employer if the person is employed. After the waiting period, sickness allowance is covered by the Social Insurance Institution of Finland (SIIF) for up to 300 compensated days. A medical certificate is required for sickness allowance.

Having a history of SA was defined as having at least one day of SA compensated by SIIF one or two years before LMT. In addition to examining all-cause SA, we also ran the analyses separately for those who received SA due to mental disorders [International Classification of Diseases (ICD-10) codes F00–F99], musculoskeletal diseases (M00–M99) and other diagnoses (the rest of the ICD-10 codes).

### Sociodemographic and work-related factors

The following variables were used in calculating the PS: age category (at five-year intervals), region of residence, place of residence in a rural or urban settlement, family structure, level and field of education, years since last education was completed, occupational class, industrial sector and employment sector (measured at the end of the calendar year preceding the year of onset of LMT). In case information on one of these variables was missing, it was replaced by information from the three previous calendar years, except for occupational class, industrial sector and employment sector which were tracked seven years before the LMT. Student status, participation in a preparatory course, or other ALMP provided by unemployment services (i.e. unemployment placement) were measured over a period of three years before LMT. In the DID analysis, we also adjusted for the duration of LMT.

### Definition of work participation

Work participation was defined based on episodes of employment, unemployment, SA, vocational rehabilitation and pensions that were available on a daily level. A person was classified as being at work if he or she was in paid employment and did not simultaneously receive any benefits or pensions. Receiving a partial SA or disability pension benefit in combination with paid employment counted as a half workday. We calculated the proportion of time spent at work based on annually measured workdays three years before the onset of LMT and three years after the end of LMT.

### Study design

We empirically grouped individuals according to their annual days of having an employment episode and of receiving unemployment benefits over three years before LMT (from here on employment history) applying k-mean cluster analysis.^20^ The analysis resulted in three clusters characterized by the level or change in employment and unemployment: (i) ‘high employment, low unemployment’ (*N* = 8172 in men, *N* = 7016 in women), (ii) ‘decreasing employment, increasing unemployment’ (*N* = 4698 in men, *N* = 4210 in women) and (iii) ‘low employment, high unemployment’ (*N* = 3324 in men, *N* = 4658 in women). A description of the derived clusters is given in Supplementary table S1.

We used PS matching to create as comparable groups as possible between LMT participants who had previous SA and those who did not. We aimed to generate two similar groups in terms of observed characteristics before the LMT and by this to control for possible baseline differences between the groups that may influence work participation after the LMT. We used logistic regression models to calculate the probability of having previous SA, with baseline characteristics as predictors. Then, within 48 strata, i.e. groups that consist of a combination of variables, sex (male, female), age group (25–44, 45–59 years old), start year of the LMT (2008–2009, 2010–2011, 2012–2013, 2014–2015) and employment history (clusters 1, 2 and 3), we applied 1:1 nearest neighbour matching without replacement to match individuals on the probability of having SA 1–2 years before LMT with a calliper of 0.1 as the maximum tolerance for matching and examined the covariate balance between the PS-matched groups.^21–25^

**Figure 1:**
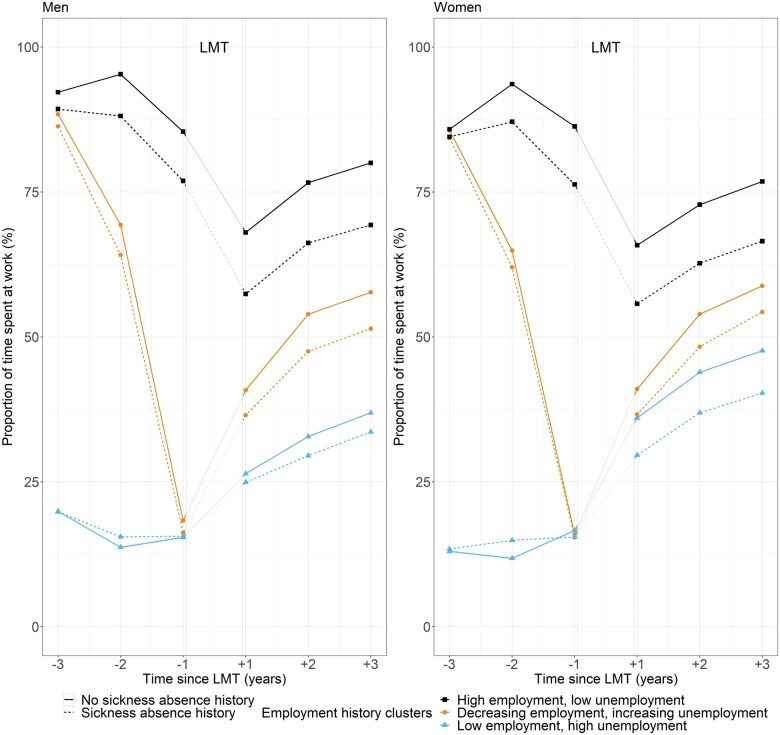
Work participation before and after vocational labour market training (LMT) by sex, employment history cluster, and sickness absence history

We analysed differences in the changes in the work participation three years before and after LMT using the DID method. The parallel assumption was tested by visually comparing the trends before and after LMT (figure 1). The DID method further controls for the differences in the unobserved time-invariant characteristics of the participants. This provides even more reliable estimates of the possible contribution of SA on work participation after LMT. All the analyses were conducted by sex and employment history. We used Stata v.16 and Psmatch2 package for the propensity score matching.^26^

### Sensitivity analyses

We further adjusted for municipal-level unemployment rates^27^ after LMT to control if the results in the main analysis were driven by the macroeconomic situation during the follow-up.

## Results

In the study population before matching, 21.3% had previous sickness absence (SA) (Supplementary table S2). Of the eligible 16 602 and 59 302 persons with and without a SA history, respectively, 16 039 pairs remained after PS matching. Matching was therefore completed for 98.9% of those who had a SA history. The differences in the baseline characteristics and the PSs between the matched and unmatched populations were small (table 1 and Supplementary table S3). These results indicate that we managed to achieve a good balance in the baseline characteristics between those with and those without a SA history. This was further supported by the results of the logistic regression models on having previous SA (Supplementary table S4). The results of the balancing test (Supplementary figure S1) indicated that PS matching improved the comparability of those with and those without SA history.

**Table 1:** Descriptive statistics of the study population consisting of participants in vocational labour market training (LMT) after matching

	After matching				
	No sickness absence history		Sickness absence history		
	*N*	%^¹^	*N*	%	chi^2^
Total	16 039	*100*	16 039	*100*	
**Sex**					*P* > 0.05
Men	8097	*50.5*	8097	*50.5*	
Women	7942	*49.5*	7942	*49.5*	
**Age category**					*P* > 0.05
25–29	1254	*7.8*	1221	*7.6*	
30–34	2558	*15.9*	2562	*16.0*	
35–39	2681	*16.7*	2676	*16.7*	
40–44	3057	*19.1*	3091	*19.3*	
45–49	3313	*20.7*	3251	*20.3*	
50–55	2904	*18.1*	2944	*18.4*	
56–59	272	*1.7*	294	*1.8*	
**Family structure**					*P* > 0.05
Single, no children	3434	*21.4*	3407	*21.2*	
Couple, children	3164	*19.7*	3103	*19.3*	
Couple, no children	6703	*41.8*	6709	*41.8*	
Single, children	1623	*10.1*	1698	*10.6*	
Other	1115	*6.9*	1122	*7.0*	
**Education**					*P* > 0.05
Primary	3076	*19.2*	3055	*19.0*	
Secondary without high school	7847	*48.9*	7734	*48.2*	
Secondary with high school	1423	*8.9*	1452	*9.0*	
Tertiary	3693	*23.0*	3798	*23.7*	
**Field of education**					*P* > 0.05
Generic programmes	3505	*21.9*	3493	*21.8*	
Education	63	*0.4*	70	*0.4*	
Arts and humanities	608	*3.8*	621	*3.9*	
Social sciences, journalism and information	89	*0.6*	91	*0.6*	
Business, administration and law	2447	*15.3*	2402	*15.0*	
Natural sciences, mathematics and statistics	78	*0.5*	75	*0.5*	
Information and Communication Technologies (ICT)	611	*3.8*	639	*4.0*	
Engineering, manufacturing and construction	4890	*30.5*	4813	*30.0*	
Agriculture, forestry, fishery and veterinary	523	*3.2*	507	*3.2*	
Health and welfare	1122	*7.0*	1171	*7.3*	
Services	2075	*12.9*	2125	*13.2*	
Unknown	28	*0.2*	32	*0.2*	
**Years since last education**					*P* > 0.05
0–10	4256	*26.5*	4215	*26.3*	
11–20	4910	*30.6*	4962	*30.9*	
21–30	4645	*28.9*	4674	*29.1*	
31–44	2223	*13.9*	2185	*13.6*	
Missing	5	*0.0*	<5	*0.0*	
**Socioeconomic status**					*P* > 0.05
Upper non-manual employees	1551	*9.7*	1571	*9.8*	
Lower non-manual employees	2140	*12.7*	2124	*13.2*	
Skilled manual workers	9903	*61.7*	9799	*61.1*	
Unskilled manual workers	1946	*12.1*	1949	*12.1*	
Self-employed	599	*3.7*	596	*3.7*	
**Industrial sector**					*P* > 0.05
Primary production^a^	343	*21.0*	347	*2.2*	
Manufacturing	4804	*29.9*	4718	*29.4*	
Construction^b^	1437	*9.0*	1364	*8.5*	
Wholesale and retail trade	1645	*10.3*	1676	*10.4*	
Transportation and storage	1087	*6.8*	1084	*6.8*	
Accommodation and food service activities	550	*3.3*	552	*3.4*	
Knowledge^c^	1419	*8.8*	1478	*9.2*	
Administrative and support service activites	1459	*9.1*	1473	*9.2*	
Public administration and defence	340	*2.1*	349	*2.2*	
Education	377	*2.3*	384	*2.4*	
Human health and social work activities	1981	*12.3*	2022	*12.6*	
Other^d^	597	*3.7*	592	*3.7*	
**Employment sector**					*P* > 0.05
Private	12 989	*80.1*	12 917	*80.5*	
Public	2476	*15.4*	2541	*15.8*	
Self-employed	574	*3.6*	581	*3.6*	
**Income category, €**					*P* > 0.05
0–12 499	2272	*14.2*	2307	*14.4*	
12 500–18 899	4338	*27.0*	4291	*26.7*	
18 900–24 799	5081	*31.7*	5073	*31.6*	
24 800–>	4348	*27.1*	4368	*27.2*	
**Region**					*P* > 0.05
Uusimaa (capital area)	3383	*21.1*	3379	*21.1*	
South	2687	*16.7*	2705	*16.8*	
West	5793	*36.1*	5761	*35.9*	
East	1991	*12.4*	1983	*12.4*	
North	2185	*13.6*	2211	*13.8*	
**Residence**					*P* > 0.05
Rural	2612	*16.3*	2609	*16.3*	
Urban	13 389	*83.5*	13 385	*83.4*	
Missing	38	*0.2*	45	*0.3*	
**Employment history cluster**					*P* > 0.05
High employment, low unemployment	7594	*47.3*	7594	*47.3*	
Decreasing employment, increasing unemployment	4454	*27.8*	4454	*27.8*	
Low employment, high unemployment	3991	*24.9*	3991	*24.9*	
**Start year of LMT**					*P* > 0.05
2008	2801	*17.5*	2818	*17.6*	
2009	3133	*19.5*	3116	*19.4*	
2010	2666	*16.6*	2642	*16.5*	
2011	1967	*12.3*	1991	*12.4*	
2012	1854	*11.6*	1871	*11.7*	
2013	1763	*11.0*	1746	*10.9*	
2014	1261	*7.9*	1243	*7.7*	
2015	594	*3.7*	612	*3.8*	
**Preparatory training**					
1 year before: yes	364	*2.3*	353	*2.2*	*P* > 0.05
1 year before: no	15 675	*97.7*	15 686	*97.8*	
2 years before: yes	427	*2.7*	403	*2.5*	*P* > 0.05
2 years before: no	15 612	*97.3*	15 636	*97.5*	
3 years before: yes	820	*5.1*	796	*5.0*	*P* > 0.05
3 years before: no	15 219	*95.9*	15 243	*95.0*	
**Unemployment placement, any**					
1 year before: yes	1350	8.4	1341	8.4	*P* > 0.05
1 year before: no	14 689	91.6	14 698	91.6	
2 years before: yes	1254	7.9	1242	7.7	*P* > 0.05
2 years before: no	14 775	92.1	14 797	92.3	
3 years before: yes	1198	7.5	1214	7.6	*P* > 0.05
3 years before: no	14 841	92.5	14 825	92.4	
**Student status, at least once 3 years before LMT**					*P* > 0.05
Was a student	8492	52.9	8466	52.8	
Was not a student	7547	47.0	7573	47.2	
*Not used in matching*					
**Duration of LMT (days)**					*P* > 0.05
0–32	4018	*25.0*	3996	*24.9*	
33–180	5348	*33.3*	5436	*33.9*	
Over 180	6673	*41.6*	6607	*41.2*	

¹ % = The proportion of individuals by background characteristics.

a Combination of agriculture, forestry and fishing; Mining and quarrying.

b Combination of electricity, gas, steam and air conditioning supply; Water supply, sewerage, waste management and remediation activities and construction.

c Combination of information and communication; Financial and insurance activities; Real estate activities; Professional, scientific and technical activities.

d Combination of arts, entertainment and recreation; Other service activities; Activities of households as employers; Undifferentiated goods- and services-producing activities of households for own use; Activities of extraterritorial organizations and bodies; Industry unknown.

### Descriptive characteristics

The matched vocational labour market training (LMT) participants were most often 40–55 years old, lived with a partner but without children, had a secondary-level education without a high school diploma, and had completed an education in the field of engineering, manufacturing or construction, and had 11–20 years since the last completed education. Typically, they were skilled manual workers, worked in the manufacturing industry and in the private sector, had an annual income of 18 900–24 799 Euros, lived in Western Finland and in an urban area, and had high employment levels before LMT, did not participate in preparatory training or other ALMPs, and were not studying before LMT. Most had an LMT that lasted for 33–180 days. The differences in the distributions of baseline characteristics between those with and those without SA history were small and not statistically significant (table 1).

### Work participation before and after LMT

Irrespective of previous SA, work participation dropped as the LMT approached and increased immediately after LMT ended, except for those who had high employment levels before LMT (figure 1). Among them, compared to one year before the LMT, work participation continued to decrease one year after LMT ended, and started to increase only two years after LMT. However, work participation remained high in this employment history cluster, although at a lower level than before LMT. Those with low employment and high unemployment before LMT had the lowest work participation both before and after LMT, although in women the increase in work participation was largest in this employment history cluster.

Those with a SA history spent consistently lower work participation after LMT than those without a SA history (figure 1). Among those with high or increasing unemployment history, the differences in work participation by SA history were small to non-existent one year before LMT but grew two to three years after LMT. The differences by SA history were the most pronounced already before LMT among those who had high employment before LMT.

The results of the age- and LMT duration-adjusted DID analyses showed that having a history of SA negatively contributed to work participation after LMT (table 2). In men, a history of SA contributed to 1.7–2.2 percentage points smaller gain in work participation one year after LMT, and in women 0.2–5.3 percentage points, depending on the employment history. Three years after LMT, the negative impact of SA history on work participation was 1.7–4.3 percentage points in men and 0.2–6.2 percentage points in women.

**Table 2:** Difference-in-differences in work participation in relation to vocational labour market training (LMT) between those with and those without a history of all-cause sickness absence history and sickness absence due to mental disorders by sex and employment history cluster. Coefficients with 95% confidence intervals

	Men			Women		
Time of measurement (ref. 1 year before the start of LMT)	**High empl., low unempl.** ^a^	Decreasing empl., increasing unempl.	Low empl., high unempl.	High empl., low unempl.	Decreasing empl., increasing unempl.	Low empl., high unempl.
**All-cause sickness absence**						
1st year after the end of LMT	−2.03*	−2.23	−1.68	−0.15	−3.91**	−5.34***
	[−3.85, −0.20]	[−4.63, 0.18]	[−4.51, 1.15]	[−2.20, 1.91]	[−6.44, −1.37]	[−7.89, −2.80]
2nd year after the end of LMT	−1.74	−4.30**	−3.37***	−0.23	−5.16***	−5.94***
	[−3.53, 0.48]	[−6.86, −1.73]	[−6.44, −0.30]	[−2.25, 1.80]	[−7.88, −2.45]	[−8.68, −3.19]
3rd year after the end of LMT	−2.01*	−4.10*	−3.42**	−0.16	−4.09**	−6.24***
	[−3.75, −0.28]	[−6.69, −1.50]	[−6.57, −0.26]	[−2.13, 1.80]	[−6.82, −1.37]	[−9.02, −3.45]
**Sickness absence due to mental disorders**						
1 year after the end of LMT	−16.57***	−9.79***	−7.70***	−7.09***	−3.56*	−4.90**
	[−19.65, −13.49]	[−12.76, −6.81]	[−10.91, −4.50]	[−9.89, −4.28]	[−6.60, −0.52]	[−7.69, −2.10]
2 years after the end of LMT	−16.32***	−9.58***	−11.39***	−6.23***	−4.44**	−6.16***
	[−19.60, −13.05]	[−12.99, −6.18]	[−15.03, −7.75]	[−9.07, −3.37]	[−7.77, −1.11]	[−9.34, −2.97]
3 years after the end of LMT	−14.05***	−7.35***	−12.72***	−6.28***	−4.23*	−5.65**
	[−17.32, −10.78]	[−10.87, −3.83]	[−16.54, −8.88]	[−9.06, −3.50]	[−7.63, −0.82]	[−8.92, −2.38]

* *P* < 0.05,

** *P* < 0.01,

*** *P* < 0.001.

Adjusted for age and LMT duration category.

a empl., employment, unempl., unemployment.

The results varied greatly by employment history. In general, a history of SA had a small (in men) to non-existent (in women) contribution to work participation among those with high employment before LMT. In men, the strongest negative contribution of SA history to work participation was among those who had increasing levels of unemployment before LMT. The increase in work participation 2–3 years after LMT was over 4 percentage points smaller among those with SA history. In women, the strongest contribution of SA history was among those with high unemployment history with a 5.9–6.2 smaller increase in work participation 2–3 years after LMT.

### Diagnosis-specific results

**Figure 2:**
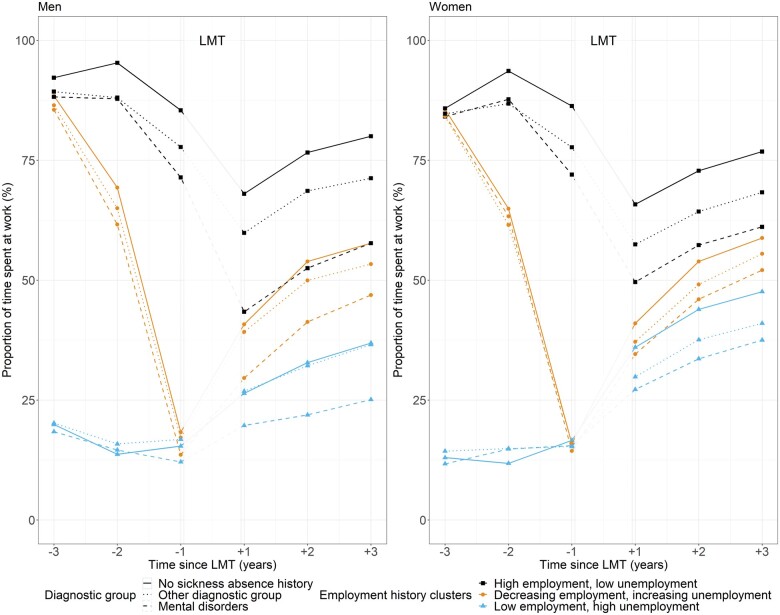
Work participation before and after vocational labour market training (LMT) by sex, employment history cluster, and sickness absence history (no sickness absence, sickness absence due to mental disorders, sickness absence due to other diagnoses)

Having a history of SA due to mental disorders led to a considerably smaller gain in work participation after LMT, especially among men and those with high employment history (figure 2 and table 2). Among men in this employment history cluster, those with SA history due to mental disorders had 14.0–16.6 percentage points, corresponding to 51–61 calendar days per year, lower increase in work participation 1–3 years after LMT than those without SA. Among women, the corresponding numbers were between 6.2 and 7.7 percentage points, i.e. 23–28 calendar days per year.

The results remained similar when analysing those who had a SA history due to mood disorders (F30–F39) or anxiety, dissociative, stress-related, somatoform and other non-psychotic disorders (F40–F48) (Supplementary figure S2). Having history of SA due to musculoskeletal diseases or other diagnoses had similar, but much weaker contributions to work participation (Supplementary figure S3).

Lastly, we analysed whether adjusting for the municipal unemployment rate affects the results, but it only had a negligible effect on the results (Supplementary table S5).

## Discussion

To the best of our knowledge, this is the first study assessing the contribution of previous work disability on work participation after vocational labour market training (LMT), or any ALMP. We used a nationwide register-based dataset and applied PS matching and DID analysis to investigate the gain in the annual proportion of time spent at work after participating in LMT among those with and those without sickness absence (SA) history among Finnish job seekers. We found that having SA one or two years before LMT overall had a negative impact on work participation after LMT, but the magnitude varied by sex, employment history, follow-up time and the diagnosis of SA. The negative contribution of SA history to work participation was stronger in women, except if the SA was due to mental disorders.

Previous work disability can influence labour market attachment after LMT in two main ways. First, it can deteriorate the success of the LMT, if the individual is unable to fully attend the training due to health problems. In the Finnish system, a job seeker must go through an application process to get into the training. As a part of this process, unemployment services evaluate the fitness and work ability of the applicant, but in practice, work disability can go unnoticed in the unemployment services.^2^^,^^4^^,^^5^ Work disability has been shown to have long-term effects that can affect labour market attachment even years later.^28^^,^^29^ Second, a history of receiving work disability benefits can also reflect a relatively good attachment to the labour market, which in turn, can improve work participation after the LMT.

Our results showed that having a history of SA is associated with poorer work participation outcomes, indicating that work disabilities are either unrecognized or otherwise not appropriately accounted for when the job seekers are offered different employment measures. The contribution of SA on work participation was particularly pronounced if the SA was due to mental disorders, resulting in 51–61 and 23–28 workdays per year smaller gain in men and women, respectively. These results support previous findings showing that SA due to mental disorders is associated with a higher risk of exit from the labour market, for example, due to disability retirement^30^^,^^31^ and unemployment.^31^ We found that the effect was especially strong in men. It is possible, that men with SA due to mental disorders suffer from more pronounced or longer-term mental disorders due to, for example, barriers related to help-seeking.^32^

The findings of this study also confirm that high employment before LMT is associated with a better work participation outcome.^12–14^ These results held despite SA history, indicating that good labour market attachment can protect from the negative effect of SA on employment or re-employment. However, the differences in work participation between those with and those without SA history were the largest among those with high employment history. It is possible, that those with more unemployment history are a more homogeneous group in terms of work disability which is relatively common among the unemployed.^1–3^ Further, while we generally observed an increase in work participation after LMT, among those with high employment before LMT this effect was delayed by one year. Positive employment effects of LMT are typically seen 1–2 years after training due to a ‘lock-in effect’ (i.e. LMT participants spend less effort on job searching during the LMT than non-participants).^13^ It is also possible, that those who had low employment before LMT were more likely active in job searching and running out of subsidized unemployment benefit days than those with high employment history.

Many countries spend a considerable amount of public finances in ALMPs. Finland’s spending on ALMP is higher than the OECD average, and a large part of it is spent on training, including LMT.^14^ While the impact of LMT on employment outcomes is positive,^4–8^ there is still room for better targeting. Supporting training among job seekers is a key factor in promoting better mental health among the population according to OECD,^33^ but at the same time, it has been recognized that public employment services offer limited access to support for individuals with mental health conditions. Particular attention should be paid to individuals with previous work disability due to mental disorders, especially due to increasing prevalence of mental disorders across Europe.^34^

### Strengths and limitations

The strengths of this study are the large, nationally representative register data with no missing data, non-response bias or drop to follow-up. Detailed data on employment and sociodemographic factors enabled us to stratify the analyses by sex and employment history. A relatively long follow-up time of three years provided a comprehensive understanding on work participation after LMT and extended beyond the period of possible lock-in effect.^13^ The use of PS matching controlling for the differences in selection due to SA is also a strength in this study. Some differences, however, remained in work participation between SA and non-SA groups among those who had high employment before LMT, indicating that the matching process was not completely successful in this subgroup. Using DID analysis partially helped to overcome this limitation. Unfortunately, we were unable to include explanatory variables such as health or lifestyle factors. Furthermore, we only included individuals who started LMT, which is only one of many possible ALMPs and it has a rather restricted entrance prevalence.^12^ We also did not have a comparison group of unemployed who did not participate in LMT; thus, the results should not be interpreted as an indication of the effectiveness of the LMT. Lastly, we were not able to assess the reason for why previous work disability would deteriorate the success of the LMT. Whether (and why) job seeker’s work disability history goes unnoticed in public employment services or whether they are allocated to LMT despite their background should be further investigated.

## Supplementary Material

ckad154_Supplementary_Data

## Data Availability

Based on the contracts with the Finnish registries, the data are not publicly available. The original register data can be accessed by contacting Statistics Finland (https://www.stat.fi/tup/mikroaineistot/index_en.html) and Findata (https://findata.fi/en/permits/data-permits/).
