# Supplementary material for: Does previous sickness absence affect work participation after vocational labour market training? A difference-in-differences propensity score matching approach
Source: Eur J Public Health. 2023 Aug 26;33(6):1071–9. doi: 10.1093/eurpub/ckad154 (PMC10710360; doi:10.1093/eurpub/ckad154)
Supplement: ckad154_Supplementary_Data [file ckad154_supplementary_data.zip › ckad154_Supplementary_Data/ejph-2023-06-om-0279-File004.docx]

Supplementary Table 1 Descriptive statistics of employment history clusters after matching. Proportion (%) of time spent in employment and unemployment during the year in the period before vocational labour market training (LMT).

|  |  | Men | |  | | Women |  |
| --- | --- | --- | --- | --- | --- | --- | --- |
|  |  | Employment | Unemployment |  | Employment | Unemployment |  |
|  |  | % | % | N | % | % | N |
| **Cluster 1 ”High employment, low unemployment”** | |  | | 8172 |  | | 7016 |
| Time before LMT | -3 years | 90.72 | 2.79 |  | 85.15 | 3.42 |  |
|  | -2 years | 91.71 | 1.77 |  | 90.37 | 1.74 |  |
|  | -1 year | 81.17 | 8.14 |  | 81.32 | 6.71 |  |
| **Cluster 2 ”Decreasing employment, increasing unemployment”** | |  | | 4698 |  | | 4210 |
| Time before LMT | -3 years | 87.36 | 4.21 |  | 84.95 | 4.46 |  |
|  | -2 years | 66.73 | 15.72 |  | 63.46 | 14.90 |  |
|  | -1 year | 17.21 | 64.68 |  | 15.66 | 59.34 |  |
| **Cluster 3 ”Low employment, high unemployment”** | |  | | 3324 |  |  | 4658 |
| Time before LMT | -3 years | 19.85 | 44.28 |  | 13.22 | 38.25 |  |
|  | -2 years | 14.63 | 50.51 |  | 13.37 | 39.26 |  |
|  | -1 year | 15.49 | 59.51 |  | 16.09 | 49.61 |  |

Supplementary Table 2. Descriptive statistics of the study population consisting of participants in vocational labour market training (LMT) before matching.

|  | Before matching | | | |  |
| --- | --- | --- | --- | --- | --- |
|  | No sickness absence history | | Sickness absence history | |  |
|  | N | % | N | % | *Chi^2^* |
| Total | 59302 | *100.0* | 16062 | *100.0* |  |
|  |  |  |  |  |  |
| **Sex** |  |  |  |  | *p<0.001* |
| Men | 34502 | *58.2* | 8106 | *50.5* |  |
| Women | 24800 | *41.,8* | 7956 | *49.5* |  |
| **Age category** | |  |  |  | *p<0.001* |
| 25–29 | 5718 | *9.6* | 1221 | *7.6* |  |
| 30–34 | 10960 | *18.5* | 2562 | *16.0* |  |
| 35–39 | 10380 | *17.5* | 2678 | *16.7* |  |
| 40–44 | 10882 | *18.4* | 3098 | *19.3* |  |
| 45–49 | 10836 | *18.3* | 3254 | *20.3* |  |
| 50–55 | 9475 | *16.0* | 2955 | *18.4* |  |
| 56–59 | 1051 | *1.8* | 294 | *1.8* |  |
| **Family structure** | |  |  |  | *p<0.001* |
| Single, no children | 12604 | *21.3* | 3413 | *21.2* |  |
| Couple, children | 11027 | *18.6* | 3108 | *19.4* |  |
| Couple, no children | 26288 | *44.3* | 6712 | *41.8* |  |
| Single, children | 4611 | *7.8* | 1707 | *10.6* |  |
| Other | 4772 | *8.0* | 1122 | *7.0* |  |
| **Education** |  |  |  |  | *p<0.001* |
| Primary | 8747 | *14.7* | 3062 | *19.1* |  |
| Secondary w/ high school | 25395 | *42.8* | 7748 | *48.2* |  |
| Secondary w high school | 5893 | *9.9* | 1452 | *9.0* |  |
| Tertiary | 19267 | *32.5* | 3800 | *23.7* |  |
| **Field of education** | |  |  |  | *p<0.001* |
| Generic programmes | 10620 | *17.9* | 3500 | *21.8* |  |
| Education | 261 | *0.4* | 70 | *0.4* |  |
| Arts and humanities | 3050 | *5.1* | 621 | *3.9* |  |
| Social sciences, journalism and information | 555 | *0.9* | 91 | *0.6* |  |
| Business, administration and law | 9098 | *15.3* | 2402 | *15.0* |  |
| Natural sciences, mathematics and statistics | 570 | *1.0* | 77 | *0.5* |  |
| Information and Communication Technologies (ICT) | 3703 | *6.2* | 639 | *4.0* |  |
| Engineering, manufacturing and construction | 19903 | *33.6* | 4813 | *30.0* |  |
| Agriculture, forestry, fishery and veterinary | 1993 | *3.4* | 507 | *3.2* |  |
| Health and welfare | 2982 | *5.0* | 1182 | *7.4* |  |
| Services | 6464 | *10.9* | 2128 | *13.2* |  |
| Unknown | 103 | *0.2* | 32 | *0.2* |  |
| **Years since last education** | | |  |  | *p<0.001* |
| 0–10 | 18115 | *30.5* | 4223 | *26.3* |  |
| 11–20 | 18968 | *32.0* | 4696 | *29.2* |  |
| 21–30 | 15633 | *26.4* | 4681 | *29.1* |  |
| 31–44 | 6573 | *11.1* | 2186 | *13.6* |  |
| Missing | 13 | *0.0* | <5 | *0.0* |  |
| **Socioeconomic status** | |  |  |  | *p<0.001* |
| Upper non-manual employees | 9347 | *15.8* | 1573 | *9.8* |  |
| Lower non-manual employees | 9057 | *15.3* | 2124 | *13.2* |  |
| Skilled manual workers | 32624 | *50.5* | 9185 | *57.2* |  |
| Unskilled manual workers | 5858 | *9.9* | 1954 | *12.2* |  |
| Self-employed | 2416 | *4.1* | 596 | *3.7* |  |
| **Industrial sector** |  |  |  |  | *p<0.001* |
| Primary production ^1^ | 1269 | *2.1* | 347 | *2.2* |  |
| Manufacturing | 17124 | *28.9* | 4719 | *29.4* |  |
| Construction ^2^ | 5515 | *9.3* | 1364 | *8.5* |  |
| Wholesale and retail trade | 6503 | *11.0* | 1676 | *10.4* |  |
| Transportation and storage | 3180 | *5.4* | 1090 | *6.8* |  |
| Accommodation and food service activities | 1768 | *3.0* | 552 | *3.,4* |  |
| Knowledge ^3^ | 7238 | *12.2* | 1478 | *9.2* |  |
| Administrative and support service activites | 5061 | *8.5* | 1476 | *9.2* |  |
| Public administration and defence | 1435 | *2.4* | 349 | *2.2* |  |
| Education | 18878 | *3.7* | 384 | *2.4* |  |
| Human health and social work activities | 5576 | *9.4* | 2034 | *12.7* |  |
| Other ^4^ | 2755 | *4.6* | 593 | *3.7* |  |
| **Employment sector** |  |  |  |  | *p<0.001* |
| Private | 48410 | *81.6* | 12929 | *80.5* |  |
| Public | 8390 | *14.1* | 2552 | *15.9* |  |
| Self-employed | 2502 | *4.2* | 581 | *3.6* |  |
| **Income category** | |  |  |  | *p<0.001* |
| 0–12,499 | 11412 | *19.2* | 2307 | *14.4* |  |
| 12,500–18,899 | 15031 | *25.3* | 4294 | *26.7* |  |
| 18,900–24,799 | 16118 | *27.2* | 5085 | *31.7* |  |
| 24,800--> | 16741 | *28.2* | 4376 | *27.2* |  |
| **Region** |  |  |  |  | *p<0.001* |
| Uusimaa (capital area) | 12281 | *20.7* | 3386 | *21.1* |  |
| South | 9534 | *16.1* | 2707 | *16.9* |  |
| West | 20256 | *34.2* | 5772 | *35.9* |  |
| East | 7685 | *13.0* | 1985 | *12.4* |  |
| North | 9556 | *16.1* | 2212 | *13.8* |  |
| **Residence** |  |  |  |  | *p<0.05* |
| Rural | 9076 | *15.3* | 2614 | *16.3* |  |
| Urban | 50067 | *84.4* | 13403 | *83.4* |  |
| Missing | 159 | *0.3* | 45 | *0.3* |  |
| **Employment history cluster** | | |  |  | *p<0.01* |
| High employment, low unemployment | 27253 | *46.0* | 7603 | *47.3* |  |
| Decreasing employment, increasing unemployment | 13354 | *22.5* | 4461 | *27.8* |  |
| Low employment, high unemployment | 18695 | *31.5* | 3998 | *24.9* |  |
| **Start year of LMT** | |  |  |  | *p<0.001* |
| 2008 | 8326 | *14.0* | 2826 | *17.6* |  |
| 2009 | 11169 | *18.8* | 3117 | *19.4* |  |
| 2010 | 10307 | *17.4* | 2644 | *16.5* |  |
| 2011 | 7914 | *13.3* | 1993 | *12.4* |  |
| 2012 | 6858 | *11.6* | 1874 | *11.7* |  |
| 2013 | 6730 | *11.3* | 1746 | *10.9* |  |
| 2014 | 5339 | *9.0* | 1248 | *7.8* |  |
| 2015 | 2659 | *4.5* | 614 | *3.8* |  |
| **Preparatory training** | |  |  |  |  |
| 1 year before: yes | 2937 | *5.0* | 796 | *5.0* | *p>0.05* |
| 1 year before: no | 56365 | *95.0* | 15266 | *95.0* |  |
| 2 years before: yes | 1778 | *3.0* | 404 | *2.5* | *p<0.01* |
| 2 years before: no | 57524 | *97.0* | 15658 | *97.5* |  |
| 3 years before: yes | 1431 | *2.4* | 353 | *2.2* | *p>0.05* |
| 3 years before: no | 57871 | *97.6* | 15709 | *97.8* |  |
| **Unemployment placement, any** |  |  |  |  |  |
| 1 year before: yes | 5679 | *9.6* | 1341 | *8.3* | *p<0.001* |
| 1 year before: no | 53623 | *90.4* | 14721 | *91.7* |  |
| 2 years before: yes | 5386 | *9.1* | 1242 | *7.7* | *p<0.001* |
| 2 years before: no | 53917 | *90.9* | 14820 | *92.3* |  |
| 3 years before: yes | 5173 | *8.7* | 1214 | *7.6* | *p<0.001* |
| 3 years before: no | 54129 | *91.3* | 14848 | *92.4* |  |
| **Student status, at least once 3 years before LMT** |  |  |  |  | *p<0.001* |
| Was a student | 34015 | *57.4* | 8472 | *52.7* |  |
| Was not a student | 25287 | 42.6 | 7590 | 47.2 |  |
|  | |  |  |  |  |
| *Not used in matching* | |  |  |  |  |
| **Duration of LMT (days)** | | | |  | *p>0.05* |
| 0–32 | 14689 | *24.8* | 3997 | *24.9* |  |
| 33–180 | 20279 | *34.2* | 5446 | *34.0* |  |
| Over 180 | 24334 | *41.3* | 6619 | *41.1* |  |
| ** p<0.05, ** p<0.01, *** p<0.001*  ^1^ Combination of Agriculture, forestry and fishing; Mining and quarrying  ^2^ Combination of Electricity, gas, steam and air conditioning supply; Water supply, sewerage, waste management and remediation activities and construction  ^3^ Combination of Financial and insurance activities; Real estate activities; Professional, scientific and technical activities  ^4^ Combination of Arts, entertainment and recreation; Other service activities; Activities of households as employers; Undifferentiated goods- and services-producing activities of households for own use; Activities of extraterritorial organisations and bodies; Industry unknown | | | | | |

Supplementary Table 3. Descriptive statistics of the unmatched population. Unmatched population with sickness absence history not reported due to low number of observations (n=23).

|  | No sickness absence history | |
| --- | --- | --- |
|  | N | *%* |
| Total | 43263 | *100.0* |
|  |  |  |
| **Sex** |  |  |
| Men | 26405 | *61.0* |
| Women | 16858 | *39.0* |
| **Age category** |  |  |
| 25–29 | 4464 | *10.3* |
| 30–34 | 8402 | *19.4* |
| 35–39 | 7699 | *17.8* |
| 40–44 | 7825 | *18.1* |
| 45–49 | 7523 | *17.4* |
| 50–55 | 6571 | *15.2* |
| 56–59 | 779 | *1.8* |
| **Family structure** |  |  |
| Single, no children | 9170 | *21.2* |
| Couple, children | 7863 | *18.2* |
| Couple, no children | 19585 | *45.3* |
| Single, children | 2988 | *6.9* |
| Other | 3657 | *8.5* |
| **Education** |  |  |
| Primary | 5671 | *13.1* |
| Secondary w/ high school | 17548 | *40.6* |
| Secondary w high school | 4470 | *6.9* |
| Tertiary | 15574 | *8.5* |
| **Field of education** |  |  |
| Generic programmes | 7115 | *16.4* |
| Education | 198 | *0.5* |
| Arts and humanities | 2442 | *5.6* |
| Social sciences, journalism and information | 466 | *1.1* |
| Business, administration and law | 6651 | *15.4* |
| Natural sciences, mathematics and statistics | 492 | *1.1* |
| Information and Communication Technologies (ICT) | 3092 | *7.1* |
| Engineering, manufacturing and construction | 15013 | *34.7* |
| Agriculture, forestry, fishery and veterinary | 1470 | *3.4* |
| Health and welfare | 1860 | *4.3* |
| Services | 4389 | *10.1* |
| Unknown | 75 | *0.2* |
| **Years since last education** |  |  |
| 0–10 | 13859 | *32.0* |
| 11–20 | 14058 | *32.5* |
| 21–30 | 10988 | *25.4* |
| 31–44 | 4350 | *10.1* |
| Missing | 8 | *0.0* |
| **Socioeconomic status** |  |  |
| Upper non-manual employees | 7796 | *18.0* |
| Lower non-manual employees | 7017 | *16.2* |
| Skilled manual workers | 22721 | *52.5* |
| Unskilled manual workers | 3912 | *9.0* |
| Self-employed | 1817 | *4.2* |
| **Industrial sector** |  |  |
| Primary production ^1^ | 926 | *2.1* |
| Manufacturing | 12320 | *28.5* |
| Construction ^2^ | 4078 | *9.4* |
| Wholesale and retail trade | 4858 | *11.2* |
| Transportation and storage | 2093 | *4.8* |
| Accommodation and food service activities | 1218 | *2.8* |
| Knowledge ^3^ | 5819 | *13.5* |
| Administrative and support service activites | 3602 | *8.3* |
| Public administration and defence | 1095 | *2.5* |
| Education | 1501 | *3.5* |
| Human health and social work activities | 3595 | *8.3* |
| Other ^4^ | 2158 | *5.0* |
| **Employment sector** |  |  |
| Private | 35421 | *81.9* |
| Public | 5914 | *13.7* |
| Self-employed | 1928 | *4.5* |
| **Income category** |  |  |
| 0–11,499 | 9140 | *21.1* |
| 12,500–17,999 | 10693 | *24.7* |
| 18,900–24,799 | 11037 | *25.5* |
| 24800--> | 12393 | *28.6* |
| **Region** |  |  |
| Uusimaa (capital area) | 8898 | *20.6* |
| South | 6847 | *15.8* |
| West | 14453 | *33.4* |
| East | 5694 | *13.2* |
| North | 7371 | *17.0* |
| **Residence** |  |  |
| Rural | 6464 | *14.9* |
| Urban | 36678 | *84.8* |
| Missing | 121 | *0.3* |
| **Employment history cluster** |  |  |
| High employment, low unemployment | 19659 | *45.4* |
| Decreasing employment, increasing unemployment | 8900 | *20.6* |
| Low employment, high unemployment | 14704 | *34.0* |
| **Start year of LMT** |  |  |
| 2008 | 5525 | *12.8* |
| 2009 | 8036 | *18.6* |
| 2010 | 7641 | *17.7* |
| 2011 | 5947 | *13.7* |
| 2012 | 5004 | *11.6* |
| 2013 | 4967 | *11.5* |
| 2014 | 4078 | *9.4* |
| 2015 | 2065 | *4.8* |
| **Preparatory training** |  |  |
| 1 year before: no | 2117 | *4.9* |
| 1 year before: yes | 41146 | *95.1* |
| 2 years before: no | 1351 | *3.1* |
| 2 years before: yes | 41912 | *96.9* |
| 3 years before: no | 1067 | *2.5* |
| 3 years before: yes | 42196 | *97.5* |
| **Unemployment placement, any** |  |  |
| 1 year before: yes | 3975 | *9.2* |
| 1 year before: no | 39288 | *90.8* |
| 2 years before: yes | 4122 | *9.5* |
| 2 years before: no | 39141 | *90.5* |
| 3 years before: yes | 4329 | *10.0* |
| 3 years before: no | 38934 | *90.0* |
| **Student status, at least once before LMT** |  |  |
| Was a student | 17740 | *41.0* |
| Was not a student | 25523 | *59.0* |
|  |  |  |
| *Not used in matching* |  |  |
| **Duration of LMT (days)** |  |  |
| 0–32 | 10667 | *24.7* |
| 33–180 | 14949 | *34.6* |
| Over 180 | 17647 | *40.8* |
| ^1^ Combination of Agriculture, forestry and fishing; Mining and quarrying  ^2^ Combination of Electricity, gas, steam and air conditioning supply; Water supply, sewerage, waste management and remediation activities and Construction  ^3^ Combination of Information and communication; Financial and insurance activities; Real estate activities; Professional, scientific and technical activities  ^4^ Combination of Arts, entertainment and recreation; Other service activities; Activities of households as employers; Undifferentiated goods- and services-producing activities of households for own use; Activities of extraterritorial organisations and bodies; Industry unknown | | |

Supplementary Table 4. Raw and fully adjusted logit models on belonging to the group with sickness absence history among participants in vocational labour market training (LMT) after matching (dichotomous variables).

|  | Fully adjusted | Raw |
| --- | --- | --- |
|  | Coefficient | Coefficient |
| **Women** (ref.men) | 0.39 | 0 |
|  |  |  |
| **Age category** |  |  |
| 25–29 | -0.14 | -0.03 |
| 30–34 | -0.12 | 0.00 |
| 35–39 | -0.13 | -0.00 |
| 40–44 | -0.11 | 0.01 |
| 45–49 | -0.13 | -0.02 |
| 50–55 | 0.08 | 0.02 |
| 56–59 | 0 | 0.08 |
| **Family structure** |  |  |
| Single, no children | -0.09 | -0.01 |
| Couple, children | -0.02 | -0.02 |
| Couple, no children | -0.00 | 0.00 |
| Single, children | 0.05 | 0.05 |
| Other | 0 | 0.01 |
| **Education** |  |  |
| Primary | -0.03 | -0.01 |
| Secondary without high school | -0.04 | -0.03 |
| Secondary with high school | -0.01 | 0.02 |
| Tertiary | 0 | 0.04 |
| **Field of education** |  |  |
| Generic programmes | -0.17 | 0.00 |
| Education | 0.08 | -0.10 |
| Arts and humanities | -0.14 | -0.02 |
| Social sciences, journalism and information | -0.17 | -0.02 |
| Business, administration and law | -0.20 | -0.02 |
| Natural sciences, mathematics and statistics | -0.22 | -0.04 |
| Information and Communication Technologies (ICT) | -0.14 | 0.05 |
| Engineering, manufacturing and construction | -0.18 | -0.02 |
| Agriculture, forestry, fishery and veterinary | -0.21 | -0.03 |
| Health and welfare | -0.13 | 0.05 |
| Services | -0.13 | 0.27 |
| Unknown | 0 | 0.13 |
| **Years since last education** |  |  |
| 0–10 | -0.61 | -0.01 |
| 11–20 | -0.63 | -0.01 |
| 21–30 | -0.63 | 0.00 |
| 31–44 | -0.59 | 0.02 |
| Missing | 0 | -0.51 |
| **Socioeconomic status** |  |  |
| Upper non-manual employees | 0.01 | -0.01 |
| Lower non-manual employees | 0.05 | -0.05 |
| Skilled manual workers | 0.03 | -0.03 |
| Unskilled manual workers | 0.03 | 0.00 |
| Self-employed | 0 | -0.00 |
| **Industrial sector** |  |  |
| Primary production ^1^ | 0.03 | 0.01 |
| Manufacturing | -0.00 | -0.02 |
| Construction ^2^ | -0.04 | -0.06 |
| Wholesale and retail trade | 0.04 | 0.02 |
| Transportation and storage | 0.00 | -0.00 |
| Accommodation and food service activities | 0.01 | 0.00 |
| Knowledge ^3^ | 0.05 | 0.04 |
| Administrative and support service activites | 0.02 | 0.01 |
| Public administration and defence | 0.02 | 0.03 |
| Education | 0.00 | 0.02 |
| Human health and social work activities | 0.01 | 0.02 |
| Other ^4^ | 0 | -0.01 |
| **Employment sector** |  |  |
| Private | 0.16** | -0.03 |
| Public | 0.25*** | 0.03 |
| Self-employed | 0 | -0.01 |
| **Income category** |  |  |
| 0–11,499 | 0.03 | 0.02 |
| 12,500–17,999 | -0.00 | -0.01 |
| 18,900–24,799 | 0.00 | -0.00 |
| 24,800--> | 0 | 0.01 |
| **Region** |  |  |
| Uusimaa (capital area) | -0.02 | -0.00 |
| South | -0.00 | 0.01 |
| West | -015 | -0.01 |
| East | -0.14 | -0.00 |
| North | 0 | 0.01 |
| **Residence** |  |  |
| Rural | -0.13 | 0.00 |
| Urban | -0.17 | -0.00 |
| Missing | 0 | 0.17 |
| **Employment history cluster** |  |  |
| High employment, low unemployment | 0.00 | 0 |
| Decreasing employment, increasing unemployment | -0.00 | 0 |
| Low employment, high unemployment | 0 | 0 |
| **Start year of LMT** |  |  |
| 2008 | -0.01 | 0.01 |
| 2009 | -0.02 | -0.01 |
| 2010 | -0.02 | -0.01 |
| 2011 | -0.00 | 0.01 |
| 2012 | -0.01 | 0.01 |
| 2013 | -0.04 | -0.01 |
| 2014 | -0.04 | -0.01 |
| 2015 | 0 | -0.03 |
| **Preparatory training** |  |  |
| 1 year before | 0.03 | 0.03 |
| 2 years before | 0.05 | -0.06 |
| 3 years before | -0.02 | -0.03 |
| **Unemployment placement, any** |  |  |
| 1 year before | 0.03 | -0.01 |
| 2 years before | 0.04 | -0.02 |
| 3 years before | -0.04 | -0.01 |
| **Student status, at least once before LMT** | -0.00 | -0.01 |
| ^1^ Combination of Agriculture, forestry and fishing; Mining and quarrying  ^2^ Combination of Electricity, gas, steam and air conditioning supply; Water supply, sewerage, waste management and remediation activities and Construction  ^3^ Combination of Information and communication; Financial and insurance activities; Real estate activities; Professional, scientific and technical activities  ^4^ Combination of Arts, entertainment and recreation; Other service activities; Activities of households as employers; Undifferentiated goods- and services-producing activities of households for own use; Activities of extraterritorial organisations and bodies; Industry unknown | | |

Supplementary Table 5. Difference-in-differences in work participation in relation to vocational labour market training (LMT) between those with and those without sickness absence history by sex and employment history after adjusting for *municipal unemployment rate*. Coefficients with 95% confidence intervals.

|  | Men | | | Women | | | |
| --- | --- | --- | --- | --- | --- | --- | --- |
| Time of measurement  (ref. 1 year before the start of LMT) | High empl.,  low unempl.^1^ | Decreasing empl.,  increasing unempl. | Low empl.,  high unempl. |  | High empl.,  low unempl. | Decreasing empl.,  increasing unempl. | Low empl.,  high unempl. |
| 1 year after the end of LMT | -2.03* | -2.25 | -1.72 |  | -0.16 | -3.90** | -5.38*** |
|  | [-3.86, -0.21] | [-4.65, 0.16] | [-4.55, 1.11] |  | [-2.21, 1.88] | [-6.44, -1.37] | [-7.92, -2.83] |
| 2 years after the end of LMT | -1.76 | -4.30** | -3.38* |  | -0.24 | -5.14*** | -5.96*** |
|  | [-3.54, 0.32] | [-6.86, -1.73] | [-6.45, -3.10] |  | [-2.27, 1.79] | [-7.85, -2.45] | [-8.70, -3.22] |
| 3 years after the end of LMT | -2.02* | -4.10** | -3.42* |  | -0.16 | -4.06** | -6.30*** |
|  | [-0.16, -0.05] | [-6.69, -1.50] | [-6.57, -0.26] |  | [-2.12, 1.80] | [-6.79, -1.33] | [-9.08, -3.51] |
| * p<0.05, ** p<0.01, *** p<0.001  ^1^ empl = employment, unempl = unemployment  Adjusted for age, LMT duration category, and municipal unemployment rate (=rate of unemployed in the municipality 1, 2, and 3 years after LMT). | | | | | | | |
