# Supplementary material for: Does previous sickness absence affect work participation after vocational labour market training? A difference-in-differences propensity score matching approach
Source: Eur J Public Health. 2023 Aug 26;33(6):1071–9. doi: 10.1093/eurpub/ckad154 (PMC10710360; doi:10.1093/eurpub/ckad154)
Supplement: ckad154_Supplementary_Data [file ckad154_supplementary_data.zip › ckad154_Supplementary_Data/ejph-2023-06-om-0279-File006.docx]

*Figure legend;*

Supplementary Figure 1. Absolute differences in the propensity scores between those with and those without sickness absence history across the variable categories before and after matching among participants in vocational labour market training (LMT).

*Footnote:*

Variables that had a smaller than 0.01-point absolute difference in the mean propensity score were excluded from this figure.

Educational field: Generic = Educational field in generic programmes

Industrial sector: Knowledge = Industrial sector in knowledge (combination of Financial and insurance activities; Real estate activities; Professional, scientific and technical activities)

Industrial sector: Human health etc. = Industrial sector in human health and social work activities

Industrial sector: Wholesale etc. = Industrial sector in wholesale and retail trade

Industrial sector: Other = Industrial sector in other (combination of Arts, entertainment and recreation; Other service activities; Activities of households as employers; Undifferentiated goods- and services-producing activities of households for own use; Activities of extraterritorial organisations and bodies; Industry unknown)

Industrial sector: Administrative etc. = Industrial sector in administrative and support service activities

Family structure: Other (other than single with or without children or living with partner with or without children)

Industrial sector: Knowledge (Combination of Information and communication; Financial and insurance activities; Real estate activities; Professional, scientific and technical activities.
