# Supplementary material for: Does previous sickness absence affect work participation after vocational labour market training? A difference-in-differences propensity score matching approach
Source: Eur J Public Health. 2023 Aug 26;33(6):1071–9. doi: 10.1093/eurpub/ckad154 (PMC10710360; doi:10.1093/eurpub/ckad154)
Supplement: ckad154_Supplementary_Data [file ckad154_supplementary_data.zip › ckad154_Supplementary_Data/ejph-2023-06-om-0279-File008.docx]

*Figure legend:*

Supplementary Figure 2. Work participation before and after vocational labour market training (LMT) by sex, employment history cluster, and sickness absence history (No sickness absence, sickness absence due to F30-F39 or F40-F48 (=mood disorders or anxiety, dissociative, stress-related, somatoform and other nonpsychotic mental disorders)).
