# Supplementary material for: Does previous sickness absence affect work participation after vocational labour market training? A difference-in-differences propensity score matching approach
Source: Eur J Public Health. 2023 Aug 26;33(6):1071–9. doi: 10.1093/eurpub/ckad154 (PMC10710360; doi:10.1093/eurpub/ckad154)
Supplement: ckad154_Supplementary_Data [file ckad154_supplementary_data.zip › ckad154_Supplementary_Data/ejph-2023-06-om-0279-File010.docx]

*Figure legend:*

Supplementary Figure 3. Work participation before and after vocational labour market training (LMT) by sex, employment history cluster, and sickness absence history (no sickness absence, sickness absence due to musculoskeletal diseases, sickness absence due to other diagnoses (=not mental disorders or musculoskeletal diseases).
