# Supplementary figures and images for: Does previous sickness absence affect work participation after vocational labour market training? A difference-in-differences propensity score matching approach
Source: Eur J Public Health. 2023 Aug 26;33(6):1071–9. doi: 10.1093/eurpub/ckad154 (PMC10710360; doi:10.1093/eurpub/ckad154)

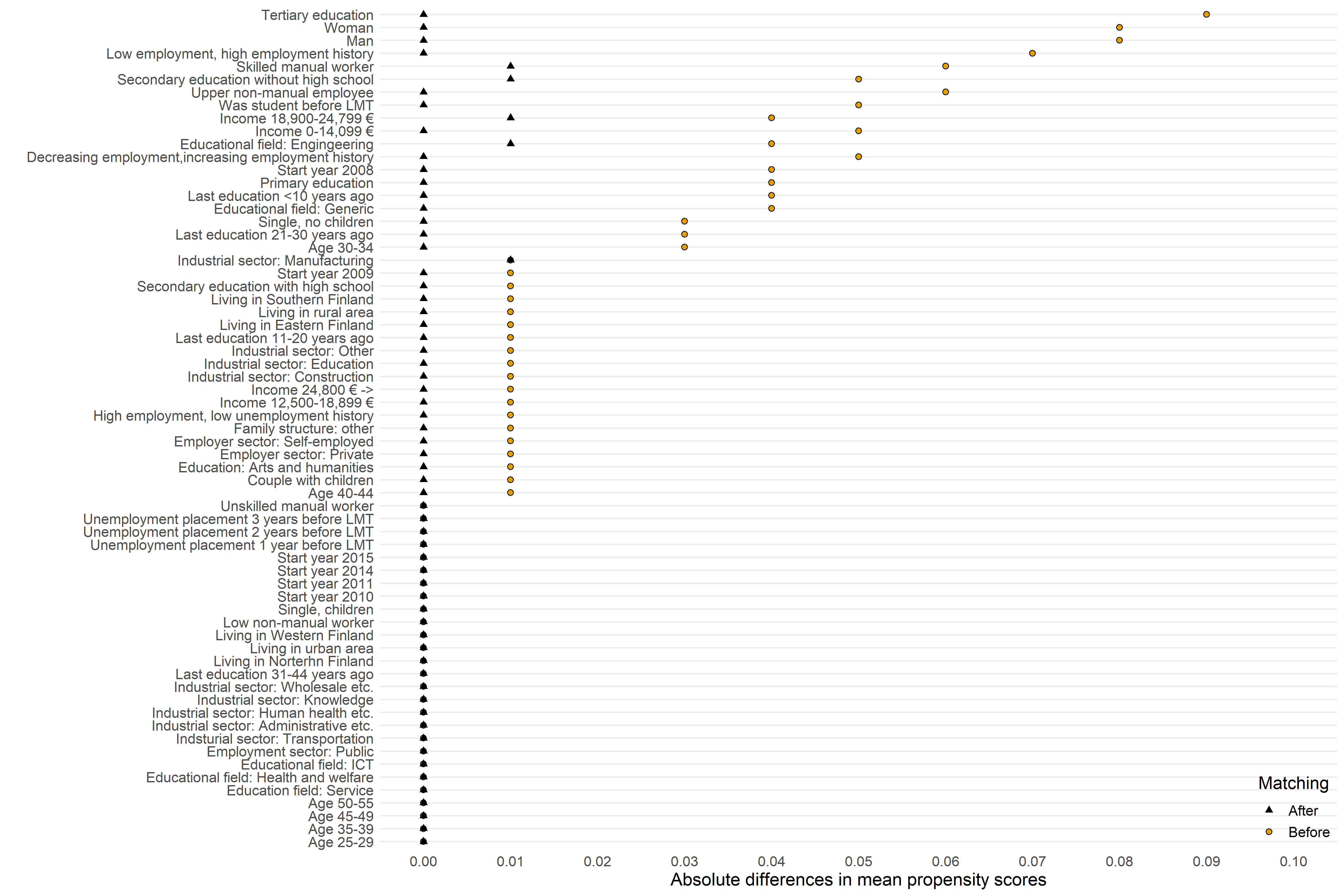

Supplement: ckad154_Supplementary_Data [file ckad154_supplementary_data.zip › ckad154_Supplementary_Data/ejph-2023-06-om-0279-File005.tiff]

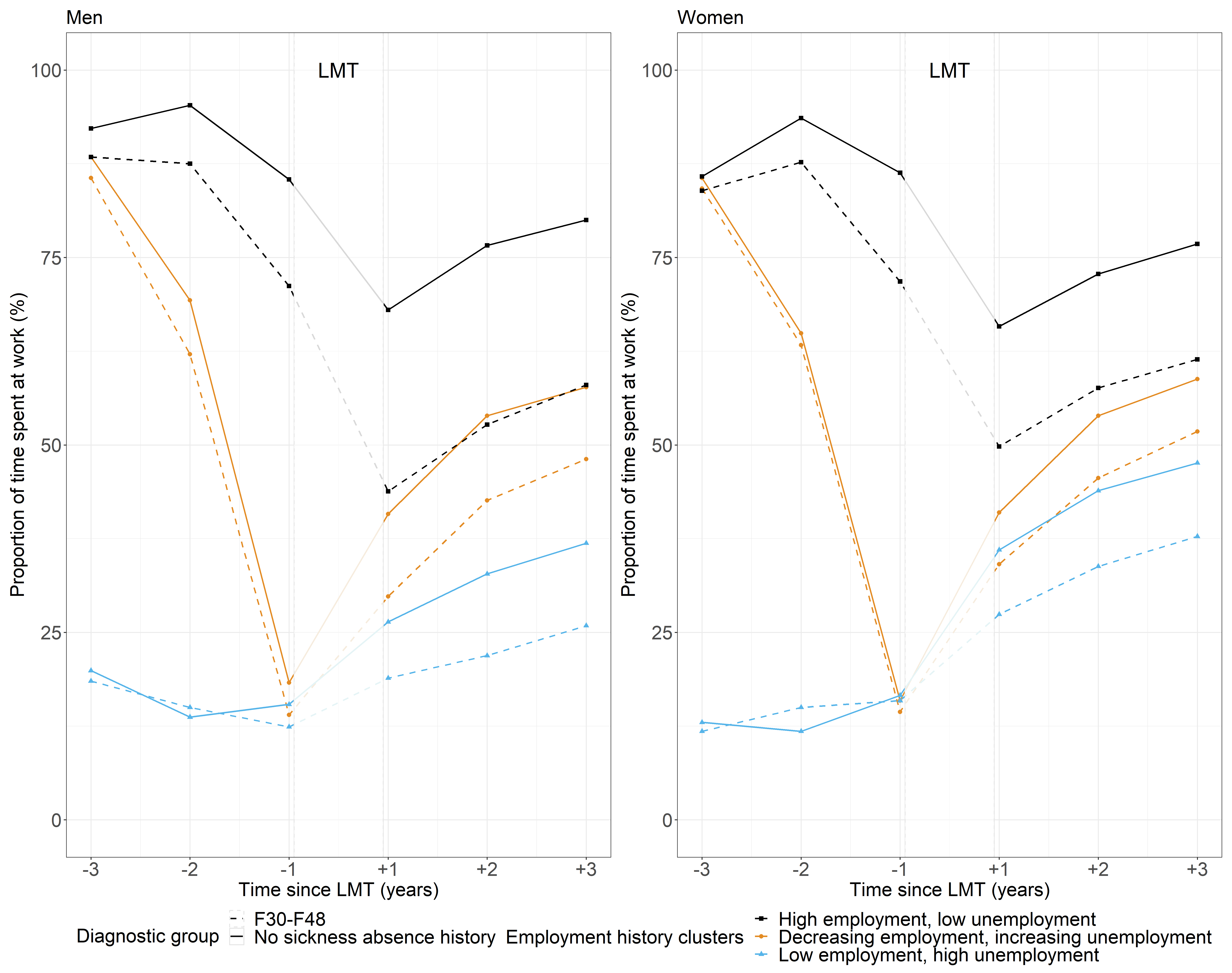

Supplement: ckad154_Supplementary_Data [file ckad154_supplementary_data.zip › ckad154_Supplementary_Data/ejph-2023-06-om-0279-File007.tiff]

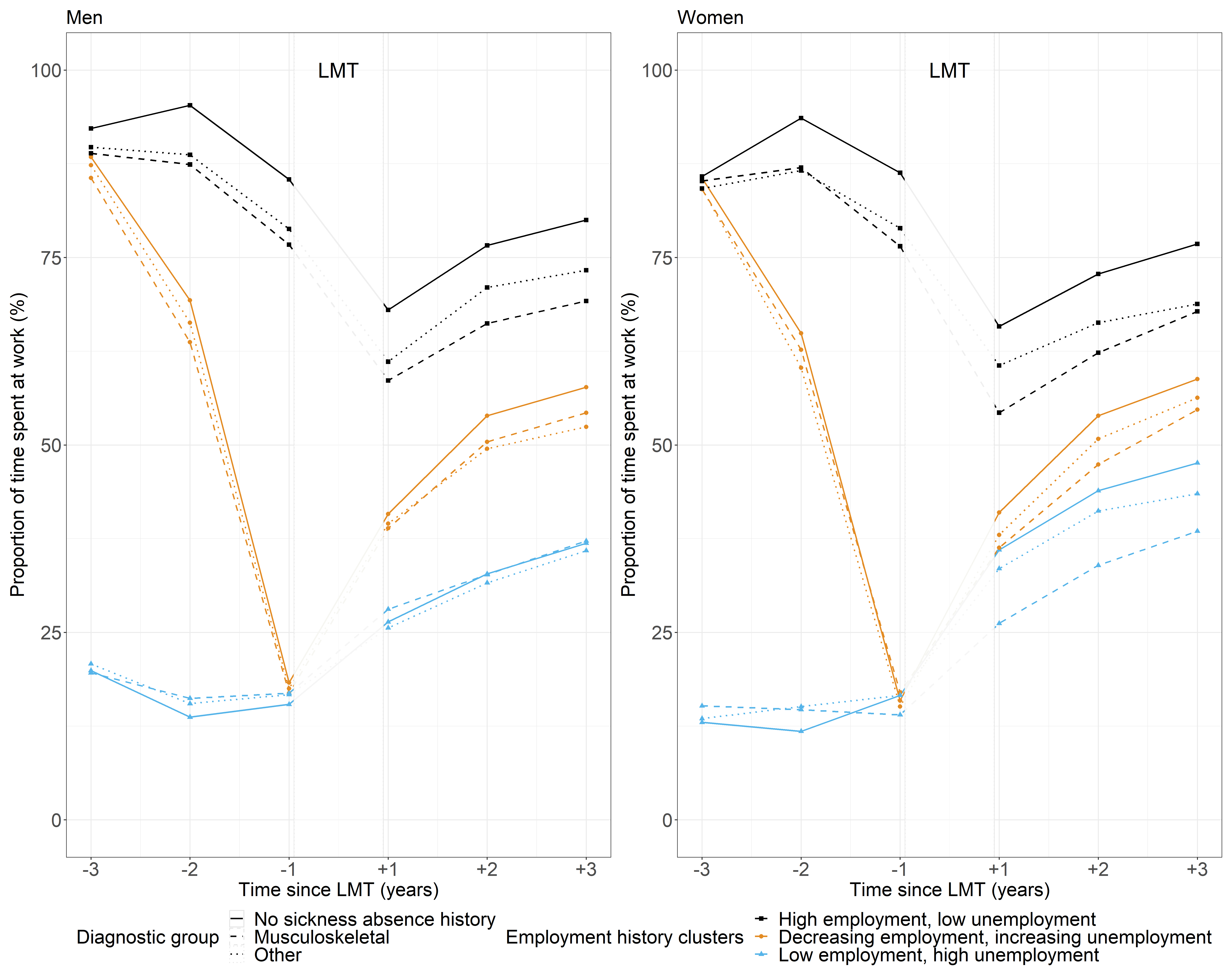

Supplement: ckad154_Supplementary_Data [file ckad154_supplementary_data.zip › ckad154_Supplementary_Data/ejph-2023-06-om-0279-File009.tiff]
